# Improving Dual-Task Walking Paradigms to Detect Prodromal Parkinson’s and Alzheimer’s Diseases

**DOI:** 10.3389/fneur.2017.00207

**Published:** 2017-05-22

**Authors:** Maroua Belghali, Nathalie Chastan, Damien Davenne, Leslie M. Decker

**Affiliations:** ^1^Normandie Université, UNICAEN, INSERM, COMETE, Caen, France; ^2^Department of Neurophysiology, Normandie Université, UNIROUEN, Rouen University Hospital-Charles Nicolle, Rouen, France

**Keywords:** gait, dual-task, attention, executive function, Alzheimer’s disease, Parkinson’s disease, prodromal phase

## Abstract

Gait control is a complex movement, relying on spinal, subcortical, and cortical structures. The presence of deficits in one or more of these structures will result in changes in gait automaticity and control, as is the case in several neurodegenerative diseases, such as Alzheimer’s disease (AD) and Parkinson’s disease (PD). By reviewing recent findings in this field of research, current studies have shown that gait performance assessment under dual-task conditions could contribute to predict both of these diseases. Such suggestions are relevant mainly for people at putatively high risk of developing AD (i.e., older adults with mild cognitive impairment subtypes) or PD (i.e., older adults with either Mild Parkinsonian signs or LRRK2 G2019S mutation). Despite the major importance of these results, the type of cognitive task that should be used as a concurrent secondary task has to be selected among the plurality of tasks proposed in the literature. Furthermore, the key aspects of gait control that represent sensitive and specific “gait signatures” for prodromal AD or PD need to be determined. In the present perspective article, we suggest the use of a Stroop interference task requiring inhibitory attentional control and a set-shifting task requiring reactive flexibility as being particularly relevant secondary tasks for challenging gait in prodromal AD and PD, respectively. Investigating how inhibition and cognitive flexibility interfere with gait control is a promising avenue for future research aimed at enhancing early detection of AD and PD, respectively.

Gait control is a complex movement involving a large class of processes, from sensory integration to movement execution and internal models of action. These processes rely on spinal, subcortical, and cortical structures (1). The presence of deficits in one or more of these structures will result in changes in gait automaticity and control, as is the case in several neurodegenerative diseases such as Alzheimer’s disease (AD) (2) and Parkinson’s disease (PD) (3).

To date, AD and PD are diagnosed in the advanced stage of degenerative brain processes, when clinical symptoms occur. In the absence of curative therapy, current research is focused on prevention by identifying subtle signs of early stage neurodegeneration. Crucially, early diagnosis would provide a critical opportunity for disease-modulating interventions targeting modifiable risk factors for AD or PD (4), or even neuro-protective therapies, both of which would help delay, slow, or even prevent disease progression [i.e., more cases would remain in the mild stage rather than degrading to moderate or severe stages (5)]. These risk factors are modifiable by making lifestyle changes (e.g., greater participation in physically and intellectually stimulating activities, social engagement, balanced diet with a high proportion of unsaturated fatty acids) and/or treating long-term health conditions (e.g., excessively high or low blood pressure, insufficient and/or fragmented sleep, diabetes, midlife obesity).

In the light of these clinical perspectives, increasing evidence supports the idea that the neuropathological process underlying PD and AD begins long before the onset of clinical symptoms as currently defined. In PD, longitudinal studies have shown that idiopathic rapid-eye-movement sleep behavior disorder (IRBD) appears 26 years before diagnosis (6), followed by anxiety (up to 20 years), and reduced gastrointestinal motility and olfaction [up to 12 years (7)]. In AD, other longitudinal studies have shown that depression appears more than 10 years before the onset of AD (8), followed by memory complaints [at least 9–10 years (9)] and mild cognitive impairment (MCI) [up to 6 years (10)]. Nonetheless, these symptoms are non-specific to these diseases, i.e., the presence of any of these symptoms does not necessarily lead to progression to these diseases. However, longitudinal data have confirmed that people with IRBD are at higher risk of conversion to PD [81% after 26 years (6)] and persons with amnesic MCI and multi-domain MCI are at higher risk of conversion to AD [80% after 6 years (10)]. Despite these findings, clinicians do not have available prodromal markers allowing them to identify MCI and IRBD patients who will develop AD or PD. In these populations, in spite of remarkable developments in neuroimaging, genetics, and molecular biomarkers, there is still an urgent need for disease-specific prodromal markers [referring to “signatures” of an ongoing pathological process prior to the presence of typical symptoms, thus allowing clinical diagnosis (11)]. Recent research focusing on such markers has already found that gait control under dual-task conditions [i.e., referring to a situation in which a secondary cognitive task is performed concurrently with a primary task while walking (12)] could contribute to predicting AD and PD in their prodromal phases (3, 13).

To summarize, both PD and AD share a long prodromal phase in which gait control under dual-task conditions is already altered. Given that the neuropathological profile of PD differs from AD, the question arises as to whether the dual-task walking paradigm is sensitive enough to identify disease-specific gait patterns. To our knowledge, this question has not been addressed in the literature. In this perspective article, we first report results supporting the idea that changes in gait control may constitute early, non-invasive, and sensitive prodromal markers of underlying neurodegeneration in PD and AD (Figure 1A). Second, we propose two dual-task models that could help specifically detect these two diseases (Figure 1B).

**Figure 1:**
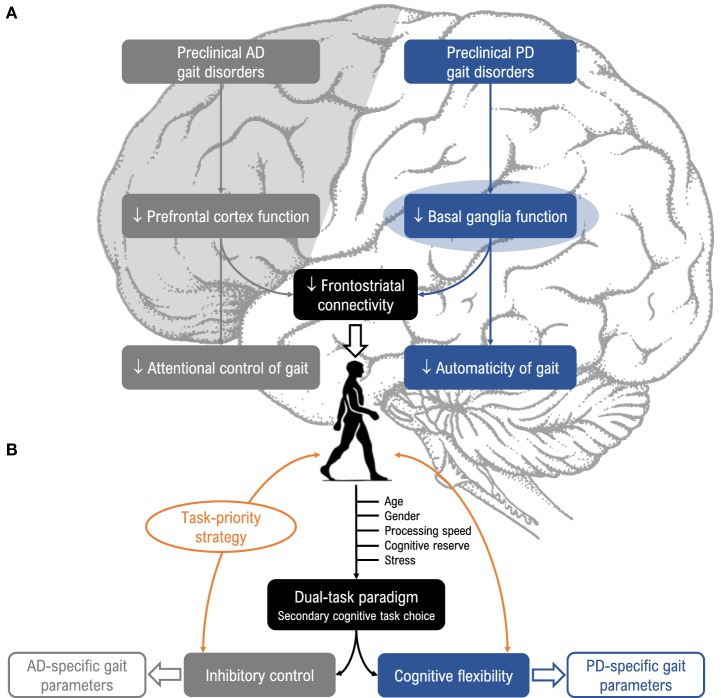
**(A)** Relevance of dual-task paradigms in detecting gait disorders in preclinical Alzheimer’s disease (AD) and Parkinson’s disease (PD). Gait disorders are characteristic features of AD (gray, left side) and PD (blue, right side) and can be unmasked under dual-task conditions. These disorders could arise from different neuropathological mechanisms in the preclinical stage, as impaired prefrontal cortex function in AD and impaired basal ganglia function in PD, and alter attentional control of gait in AD (i.e., “cortical gait disorders”) and automaticity of gait in PD (i.e., “cortical-subcortical gait disorders”). **(B)** Suggestions for choice of cognitive tasks and gait parameters. Dual-task gait assessment using targeted cognitive tasks (i.e., inhibitory control for AD, and cognitive flexibility for PD), taking into account task prioritization (trade-off effects) and factors known to modulate susceptibility to dual-task interference (age, gender, processing speed, stress, and cognitive reserve) would be valuable for enhancing detection of prodromal AD and PD.

## Dual-Task-Related Gait Changes in Prodromal PD

Parkinson’s disease is manifested by loss of dopamine-secreting neurons in the substantia nigra (14), leading to a lack of dopamine in the striatum and causing disruption of the basal ganglia circuit (15). At the behavioral level, it is widely accepted that gait deficits are among the most important motor problems associated with PD. The latter are characterized by deficits in all spatio-temporal gait parameters (16), rhythmic limb movements (17), arm swing amplitude and symmetry (18), bilateral arm coordination (19), and adjustment of postural muscle tone (20). Mechanistically, gait deficits may result from a mixture of several factors including: (i) a loss of motor automaticity mediated by impaired internal cueing mechanisms and less effective connectivity between motor areas (21), (ii) use of attentional control strategies in order to bypass impaired automatic motor processes (21), (iii) impaired motor planning mediated by decreased activity in the caudal cingulate motor area (22), (iv) axial rigidity mediated by exaggeration of long-latency reflexes (23), (v) cognitive inflexibility mediated by changes in frontal-striatal circuits (24, 25), and (vi) the inability to use a “posture-first” strategy (i.e., prioritizing gait over other concurrent tasks). The presence of non-dopaminergic pathology, such as serotonin, norepinephrine or acetylcholine, and muscle weakness may also explain gait deficits in PD (16).

In contradiction with these findings, some studies (26, 27) found that certain aspects of gait such as gait speed and step length were unexpectedly unaffected under dual-task conditions. According to the review by Bloem et al. (28), PD patients improved their gait performance by using external cues allowing the frontal cortex to compensate for the defective basal ganglia circuitry (28–30). Another possible explanation is that PD patients were able to prioritize gait to the detriment of cognitive tasks [“posture-first” strategy (28, 31)]. Recently, the study by Rochester et al. (31) suggests that the use of an auditory cognitive task may also improve the gait performance of PD patients. Such a modality may also provide beneficial cueing effects on gait rhythm through compensatory mechanisms or by facilitating task prioritization (31).

At present, the onset of PD appears after depletion of 70–80% of striatal dopamine, corresponding to 30–50% of dead cells of dopaminergic neurons (3). Clinically, gait deficits that should appear because of basal ganglia dysfunction and loss of dopamine are not detectable under undisturbed walking conditions (i.e., self-selected comfortable speed under single-task condition) in prodromal PD (32). This might be due to satisfactory compensatory mechanisms in the motor system which compensate for the slowly progressing nigrostriatal dopamine depletion, both within and outside the basal ganglia (33). It has been suggested that dual-task walking might be a valuable tool for unmasking the use of compensatory strategies (32, 34, 35). Currently, all studies (*n* = 3) conducted in the prodromal stage of PD support this view. The first study by Lerche et al. (35) showed that individuals with mild parkinsonian signs slowed down to the same extent as non-demented patients with PD when performing dual-task walking. The two other studies (34, 36) showed increasing in-stride time variability, arm swing asymmetry, and arm swing variability under dual-task walking conditions in LRRK2 G2019S mutation carriers without a clinical diagnosis of PD. To our knowledge, no study has been conducted in IRBD.

Given the limited number of studies focusing on gait in the prodromal phase of PD, the exact mechanisms underlying dual-task-related gait changes remain unclear. Nonetheless, it is likely that these changes reflect early impairment of motor automaticity due to an early dysfunction in basal ganglia–brainstem pathways (34, 35) and cognitive inflexibility (35). Thus, we suggest that frontal–striatal network deficits involved in both cognitive flexibility and gait automaticity could be considered as possible mechanisms contributing to dual-task-related gait changes in prodromal PD. Besides, we suggest that limited attentional capacities could also prevent patients from sharing attention (i.e., central capacity sharing model) and/or correctly processing the attentional demands of each task (i.e., central bottleneck model). Indeed, the central bottleneck model postulates that central processing acts on only one task at a time, and therefore, constitutes a bottleneck that processes tasks serially (37, 38), whereas the central capacity sharing model claims that the central stage is a limited-capacity parallel processor that divides resources among to-be-performed tasks (39, 40).

## Dual-Task-Related Gait Changes in Prodromal AD

Alzheimer’s disease is characterized by the presence of both amyloid beta plaques and neurofibrillary tangles [i.e., the two hallmark pathologies (41, 42)] in the medial temporal lobe structures (particularly the hippocampus) and neocortex, together with neuronal loss and volume reductions (43, 44). At behavioral level, AD is characterized by marked impairment in several cognitive domains including the episodic, working and semantic memories, visuo-spatial abilities, language, and executive attention control (45, 46). Interestingly, these cognitive impairments affect gait control (1, 47–50). From the literature, the picture emerges that deficits in cognition are manifested through subtle gait control deficits in pace (i.e., step velocity, step length, step time variability, swing time variability, stance time variability) and variability (i.e., step velocity variability, step length variability, step width variability) domains of gait (50). In addition, other studies suggest that the hyperexcitability of the motor cortex may also contribute to gait deficits in clinical AD.

An autopsy study on brains from non-demented and demented individuals (43) has shown that extracellular amyloid deposition usually appears before any intraneuronal neurofibrillary changes, first in the entorhinal cortex before spreading in a hierarchical manner into the hippocampus proper and cortex (43). A longitudinal neuropsychological study of the transition from healthy aging to AD (51) revealed that impairment in *executive attention control*, particularly *inhibition* [i.e., ability to deliberately inhibit dominant, automatic, or prepotent responses when required and/or requested (52)], precedes impairment in episodic memory in the course of the disease and, importantly, predicts AD. The recent neuroimaging study by Harrington et al. (53) has shown that abnormal extracellular amyloid accumulation in the basal isocortex impairs inhibition [as assessed by the Stroop task (54)] in asymptomatic individuals with abnormal beta-amyloid 42/tau ratios. It is worth mentioning that the underlying pathways of inhibition include the bilateral dorsolateral prefrontal cortex, inferior frontal gyrus, anterior cingulate cortex, and posterior parietal cortex (55). Collectively, these findings suggest that prefrontal dysfunction is present during the early stages (preclinical) of AD (53).

At the onset of MCI (i.e., prodromal phase of AD), the neuropathological mechanisms underlying progression to AD become more complex. Nevertheless, several studies have demonstrated reduced hippocampal and entorhinal cortex volume, as well as reduced cortical thickness in the medial and lateral temporal cortex, parietal lobe, and frontal lobes in MCI patients who have converted to AD (MCI-converters), up to 2 years prior to clinical conversion, as compared to MCI patients who remained stable (MCI-stable) (56–59). At the behavioral level, longitudinal studies found that MCI-converters performed worse than controls on measurements of episodic and working memory (60) and of executive attention control, particularly inhibition (61). Hence, it is likely that executive inhibitory control could also represent a hallmark of early AD.

Anatomically, the prefrontal cortex plays a major role not only in executive attention control but also in gait control through its connection with the striatum (62). Thus, one could hypothesize that changes in executive attention control should be reflected in gait, notably when having to devote attentional resources to a simultaneously performed secondary task. Several studies using dual-task walking paradigms support this view. Clinically, the fact that changes in executive attention control can be measured through dual-task assessment has opened up a new line of research devoted to identify a “gait phenotype” of abnormal cognitive decline due to underlying AD or other types of dementia (2). Unfortunately, to date, no studies using the dual-task walking paradigm have been conducted at the preclinical stage of AD. However, several cross-sectional studies have been performed at the prodromal stage among various MCI patient subtypes. Findings revealed that walking under dual-task conditions induced differential changes in gait velocity and variability of various spatio-temporal parameters (13, 63–67). Such changes, named “cortical gait disorders,” have been interpreted to reveal impairments in both working memory and executive attention control (67). Studies using magnetic resonance spectroscopy in MCI patients showed that dual-task-related gait changes are associated with changed neurochemistry (i.e., lower *N*-acetyl aspartate/creatine) and lower hippocampal and primary motor cortex volumes (68). Other interpretations are the central bottleneck and central capacity sharing models (37–40).

Collectively, these findings suggest that the neuropathological mechanisms underlying dual-task-related gait changes in MCI patients may result from a combination of several factors. However, recent studies support the idea that impairments in both frontal–hippocampal circuits [involved in spatial orientation and navigation (64)] and prefrontal–striatal circuits [involved in executive attention control (2)] may potentially contribute to gait deficits.

Importantly, in contrast to the above findings, two studies found a pattern of dual-task interference in MCI patients that was not significantly different from healthy older adults (69, 70) since both groups became slower and more variable during dual-task walking. Although not reported by the authors, one can speculate that not all of the MCI patients would have converted to dementia. Therefore, it remains highly plausible that the use of dual-task gait assessment could allow improved discrimination between converters and non-converters. A longitudinal study has provided evidence supporting this hypothesis (71), by showing that MCI-converters reduced gait speed and increased gait variability under dual-task conditions more than non-converters. However, additional longitudinal studies are needed to confirm these findings.

## How Could Dual-Task Gait Assessment be Improved to be Used Potentially as a Robust and Clinically Relevant Marker of Prodromal AD or PD?

As discussed above, dual-task-related gait decrements may provide a powerful prodromal marker of neurodegenerative diseases, such as AD and PD. However, the use of dual-task paradigm in clinical practice requires further clarifications on some methodological aspects. First, the type of cognitive task to be used as a concurrent secondary task has to be selected among the plurality of tasks proposed in the literature. Second, the key aspects of gait control that represent sensitive and specific “gait signatures” for prodromal AD or PD need to be determined. Third, interindividual differences in factors known to play a strong role in shaping expression and timing of onset of disease should be taken into account when analyzing dual-task performance.

### Suggestions for Future Research Directions

Dual-task-related gait changes may vary depending on the type of secondary cognitive task. Previous literature suggests that cognitive tasks involving internal interfering factors seem to disturb gait performance more than those involving external interfering factors (72). The modality of the secondary cognitive task may also influence gait performance, particularly in PD patients who are less sensitive to dual-task interference under the auditory modality. In our view, a way to make better use of dual-task walking paradigms is to select a secondary cognitive task that both interferes with gait control and challenges the underlying neuropathological processes (in favor of the bottleneck model). Since specific impairments are found in inhibition for AD (51, 53) and cognitive flexibility for PD (35), we suggest the use of a visual Stroop interference task requiring executive inhibitory control (73) and a visual set-shifting task requiring reactive flexibility (74) as particularly relevant secondary tasks for challenging gait in prodromal AD and PD, respectively. Such tasks are known to interfere with gait control (50, 75) and importantly, begin to deteriorate years prior to the onset of the first clinical symptoms. Moreover, cognitive flexibility and inhibition depend on dopamine function in the basal ganglia (76) and entorhinal cortex (77), respectively (Figure 1B). In order to reduce patient stress induced by dual-task gait assessment, virtual environments using embedded secondary cognitive tasks appear to offer a good solution due to their ecological character (78).

Regarding the choice of gait parameters, lower-limb gait parameters have been widely studied and characterized, whereas less is known about upper-limb gait parameters in both AD and PD. However, previous studies have highlighted that deficits in the latter parameters (e.g., arm swing amplitude and symmetry, bilateral arm coordination) are characteristic features of PD. Surprisingly, these upper-limb gait parameters, known to be linked to dysfunction of basal ganglia (18, 19, 79, 80), were explored in only one study in the prodromal stage of PD (36). Therefore, investigating how gait parameters related to basal ganglia dysfunction change in prodromal PD is a useful approach that should be considered in future studies.

In response to the need for theoretical development in measuring dual-task performance, Plummer et al. (81, 82) have proposed an elegant conceptual model for pattern classification of dual-task interference by assessing the interactions between two simultaneously performed tasks (i.e., trade-off strategy). In addition, we would argue that it is also imperative to take into account factors modulating the capacity to cope with a concurrent cognitive load while walking, among which the most important are age, gender, processing speed (83), stress, and cognitive reserve [i.e., the background cognitive capacity that a subject brings to a given task (84)]. Applying these methodological recommendations for the conduct of future research would likely enhance the reliability of dual-task gait assessment and its large-scale application in clinical practice.

More broadly, the only study design approach allowing identification of robust, clinically relevant gait markers for preclinical AD or PD consists in longitudinal studies aimed at comparing patients from complaints to disease expression (preclinical, prodromal, and clinical) from those who will remain stable. To date, no such studies have been conducted on patients with cognitive complaints and/or IRBD, and only one has been carried out on MCI, although without a control group.

In conclusion, we can consider dual-task-related changes in gait control, taking into account task prioritization (trade-off effects) and factors known to modulate susceptibility to dual-task interference, as potential markers for prodromal neurodegeneration. Investigating how inhibition and cognitive flexibility interfere with gait control is a promising avenue for future research aimed at enhancing early detection of AD and PD, respectively. Longitudinal studies and standardized protocols would certainly ensure consistency and aid interpretation.

## Author Contributions

MB and LMD wrote the manuscript. NC and DD revised it critically for important intellectual content. All authors approved its final version and contributed to the conceptual figure.

## Conflict of Interest Statement

The authors declare that the research was conducted in the absence of any commercial or financial relationships that could be construed as a potential conflict of interest.
